# Stricture of the Urethra

**Published:** 1900-06-16

**Authors:** 


### Hospital Clinics and Medical Progress.

### STRICTURE OF THE URETHRA.

In a clinical lecture recently delivered at St. George's Hospital Mr. William Bennett drew attention to several points in the management of stricture of the urethra which are distinctly noteworthy. Firstly, as to the advantage of small wax bougies in getting through an apparently impassable stricture. In the case before him he commenced by passing down the urethra a small wax bougie about the size of a No. 1 (English). There was some little difficulty in getting through the stricture at first, but after trying two or three of these little instruments one of the smallest possible size slipped through the constriction without any sense of resistance and without causing any pain, and it was pointed out that the facility with which such a fine wax bougie will pass through a stricture which is quite impassable by other means is something quite remarkable, it being the rarest thing for a stricture not to be passable in this way. After this small bougie had remained in the stricture for about five minutes, so as to render the parts tolerant of a foreign body, a No. 2 (English) silk bougie was passed down by its side, and, on reaching the constriction the wax bougie was gently withdrawn by the house surgeon while the silk bougie was gently pressed onwards. As the wax bougie left the stricture the silk one slipped in with perfect ease, after which the same manœuvre was repeated with No. 3 silk, and so on, until a No. 6 catheter was tied in; the whole being accomplished in one sitting without the loss of a drop of blood. After this, at intervals of three days, the size of the retained catheter was increased by stages until No. 9 was reached. The case in the end turned out to be a resilient stricture, and had to be divided by internal urethrotomy; but we go on to the next point, namely, that however freely a stricture has been divided urethral spasm may arise during the passage of an instrument unless the meatus also has been slit sufficiently to enable it to pass easily through it; in other words, the spasm which is often so troublesome a complication in the treatment of a stricture arises in many cases from irritation of the meatus rather than from irritation at the seat of the stricture. Hence, no doubt, much of the advantage which is found in some cases from the use of bulbous-ended instruments. It is not merely that the instrument is left free to turn easily without being held by the meatus, but that by being so left free absence of spasm in the deeper parts also is ensured. The next point, and, if true, a very important one, is that the deeper mischief which is so apt to be set up in the ureters and the kidneys by a stricture of the urethra, is not caused by the back-pressure resulting directly from the obstruction to the outflow to the urine so much as by that produced by the voluntary straining habitually indulged in by the patient. The amount of renal disease met with in a case of stricture is not always, he says, in direct proportion to the tightness or density of the constriction. Even such as are extremely tight are not always followed by renal changes such as sometimes ensue in strictures which are in themselves comparatively slight. The cause of this is that the one patient has strained, and the other has been content to let the urine take its time. No matter how tight a stricture may be, the bladder—in the absence, of course, of actual retention—will empty itself spontaneously without straining if it be allowed to do so, although the process may be slow and tedious, and no more back pressure will be produced than the urinary mechanism will bear without damage. If, however, the patient habitually strains to expedite the flow, the backward pressure becomes so great that dilatation of the ureter and kidneys soon commences. This is a very interesting point, although perhaps the problem may not be quite so simple a one as Mr. Bennett suggests. Lastly, Mr. Bennett states that straining may itself set up retention. It will be found on inquiry, he says, that in quite a number of cases retention has come on whilst the patient has been straining to accelerate the flow of a slow stream. This he gives as another reason for believing that there are few points of more importance in the management of stricture than the necessity for the entire avoidance of strain in passing urine; and he adds that in advanced stricture the bladder is rarely, if ever, completely emptied in patients who strain.

